# Luteolin Alleviates AflatoxinB_1_-Induced Apoptosis and Oxidative Stress in the Liver of Mice through Activation of Nrf2 Signaling Pathway

**DOI:** 10.3390/antiox10081268

**Published:** 2021-08-09

**Authors:** Shahid Ali Rajput, Aftab Shaukat, Kuntan Wu, Imran Rashid Rajput, Dost Muhammad Baloch, Rana Waseem Akhtar, Muhammad Asif Raza, Agnieszka Najda, Papliński Rafał, Ashraf Albrakati, Attalla F. El-Kott, Mohamed M. Abdel-Daim

**Affiliations:** 1 Department of Animal Nutrition and Feed Sciences, College of Animal Science, South China Agricultural University, Guangzhou 540642, China; 2 National Center for International Research on Animal Genetics, Breeding and Reproduction (NCIRAGBR), Huazhong Agricultural University, Wuhan 430070, China; dr.aftabshaukat@mail.hzau.edu.cn; 3 Department of Animal Nutrition and Feed Science, College of Animal Science and Technology, Huazhong Agricultural University, Wuhan 430070, China; kuntanwu@webmail.hzau.edu.cn; 4 Faculty of Veterinary and Animal Science, Lasbela University of Agriculture Water and Marine Science, Uthal 89250, Pakistan; drimranrajput@gmail.com; 5 Department of Biotechnology, Lasbela University of Agriculture Water and Marine Science, Uthal 89250, Pakistan; vc@luawms.edu.pk; 6 Faculty of Veterinary and Animal Sciences, Muhammad Nawaz Shareef University of Agriculture, Multan 66000, Pakistan; dr.ranawasim2@gmail.com (R.W.A.); asifrazarana@yahoo.com (M.A.R.); 7 Department of Vegetable Crops and Medicinal Plants, University of Life Sciences in Lublin, 50A Doświadczalna Street, 20-280 Lublin, Poland; agnieszka.najda@up.lublin.pl (A.N.); rafal.paplinski@up.lublin.pl (P.R.); 8 Department of Human Anatomy, College of Medicine, Taif University, P.O. Box 11099, Taif 21944, Saudi Arabia; a.albrakati@tu.edu.sa; 9 Biology Department, Faculty of Science, King Khalid University, Abha 61421, Saudi Arabia; elkottaf@kku.edu.sa; 10 Zoology Department, College of Science, Damanhour University, Damanhour 22511, Egypt; 11 Department of Pharmaceutical Sciences, Pharmacy Program, Batterjee Medical College, P.O. Box 6231, Jeddah 21442, Saudi Arabia; abdeldaim.m@vet.suez.edu.eg; 12 Pharmacology Department, Faculty of Veterinary Medicine, Suez Canal University, Ismailia 41522, Egypt

**Keywords:** aflatoxin B_1_, luteolin, oxidative stress, apoptosis, liver injury, Nrf2 signaling pathway

## Abstract

Aflatoxin B_1_ (AFB_1_), a threatening mycotoxin, usually provokes oxidative stress and causes hepatotoxicity in animals and humans. Luteolin (LUTN), well-known as an active phytochemical agent, acts as a strong antioxidant. This research was designed to investigate whether LUTN exerts protective effects against AFB_1_-induced hepatotoxicity and explore the possible molecular mechanism in mice. A total of forty-eight mice were randomly allocated following four treatment groups (*n* = 12): Group 1, physiological saline (CON). Group 2, treated with 0.75 mg/kg BW aflatoxin B_1_ (AFB_1_). Group 3, treated with 50 mg/kg BW luteolin (LUTN), and Group 4, treated with 0.75 mg/kg BW aflatoxin B_1_ + 50 mg/kg BW luteolin (AFB_1_ + LUTN). Our findings revealed that LUTN treatment significantly alleviated growth retardation and rescued liver injury by relieving the pathological and serum biochemical alterations (ALT, AST, ALP, and GGT) under AFB_1_ exposure. LUTN ameliorated AFB_1_-induced oxidative stress by scavenging ROS and MDA accumulation and boosting the capacity of the antioxidant enzyme (CAT, T-SOD, GSH-Px and T-AOC). Moreover, LUTN treatment considerably attenuates the AFB_1_-induced apoptosis in mouse liver, as demonstrated by declined apoptotic cells percentage, decreased Bax, Cyt-c, caspase-3 and caspase-9 transcription and protein with increased Bcl-2 expression. Notably, administration of LUTN up-regulated the Nrf2 and its associated downstream molecules (HO-1, NQO1, GCLC, SOD1) at mRNA and protein levels under AFB_1_ exposure. Our results indicated that LUTN effectively alleviated AFB_1_-induced liver injury, and the underlying mechanisms were associated with the activation of the Nrf2 signaling pathway. Taken together, LUTN may serve as a potential mitigator against AFB_1_-induced liver injury and could be helpful for the development of novel treatment to combat liver diseases in humans and/or animals.

## 1. Introduction

Aflatoxins are one of the most dangerous mycotoxins, produced mainly by *Aspergillus flavus* and *Aspergillus parasiticus* and usually found in agricultural environments and food commodities [1]. According to studies, even low levels of aflatoxins in the diet can be harmful to human health [2,3]. Currently, approximately 4.5 billion people are in danger of being exposed to aflatoxins, which cause 4.6–28.2% of all occurrences of hepatocellular carcinoma worldwide [4,5]. Aflatoxin B_1_ (AFB_1_) is the most potent liver carcinogen among the aflatoxins, and it has been categorized as a Class I carcinogen by the International Agency for Research on Cancer (IARC) [6]. Additionally, AFB_1_ is reported to cause severe health issues, including growth retardation, hepatotoxicity, neurotoxicity, teratogenicity, mutagenicity and immunotoxicity in humans and animals [7,8,9]. The liver is believed to be the primary target organ for AFB_1_ toxicity, a major metabolizing and detoxifying organ in the body [10]. Previous research has shown that reactive oxygen species (ROS) formation appears to be a significant contributor to the toxicity caused by AFB_1_ in the liver [11]. ROS overproduction could lead to mitochondrial oxidative stress, resulting in lipid peroxidation and decreased antioxidant enzyme activity, causing cellular and organismal hepatic damage [5,12]. Therefore, it is imperative to find an effective antioxidant to protect the liver and alleviate the AFB_1_ toxicity. Natural active biological compounds derived from plants have recently received much attention due to their low toxicity. Herbal metabolites are effective alternatives for tackling the hazardous effects of AFB_1_ [13,14].

Flavonoids are bioactive compounds primarily present in plants with a wide range of pharmacological and health-promoting properties [15]. Luteolin (3,4,5,7-tetrahydroxyflavone) is a type of natural flavonoid found in various plants worldwide, such as fruits, vegetables, and some herbal medicines [16]. Luteolin possesses a diverse range of biological properties, including antioxidant [17], antimicrobial [18], anti-inflammatory [19], anticancer [20], and neuroprotective capabilities [21]. The nuclear factor erythroid 2-related factor (Nrf2) plays a central role in the activation of cytoprotective genes in response to xenobiotics and oxidative stress by binding to the antioxidant response element (ARE) [22,23]. Moreover, the Nrf2 gene is typically expressed in metabolically active tissues such as the liver [10]. A recent study revealed that AFB_1_-induced liver damage in broiler chicks has been associated with dysregulating the Nrf2 signaling pathway [13]. Therefore, Nrf2 signaling is regarded as the most significant therapeutic target for preventing and treating oxidative stress-induced liver damage [24,25]. Although, a study reported that luteolin has a therapeutic impact on ochratoxin A-induced oxidative injury in NRK-52E kidney cells [17]. Moreover, LUTN has been reported to exert antifibrogenic effects against carbon tetrachloride-(CCL4) on hepatic satellite cells and liver fibrosis via multiple mechanisms [26,27]. However, the preventative actions of LUTN against AFB_1_-induced liver damage and underlying mechanisms have still not been explored. Therefore, the current research was designed to investigate whether LUTN exerts protective effects against AFB_1_-induced hepatotoxicity and explore the possible molecular mechanism in mice. Presumably, this is the first study to highlight the protective role of luteolin against AFB_1_-induced hepatic damage in mice.

## 2. Materials and Methods

### 2.1. Chemicals and Antibodies

The luteolin (LUTN, #41753–43-9, purity ≥ 98%) was bought from (Shanghai Yuanye Bio-Technology Co., Ltd.). Aflatoxin B_1_ (AFB_1_, #1162-65-8) used in the present study was supplied by Sigma-Aldrich (St. Louis, MO, USA). The ELISA assay kits for malondialdehyde (MDA, #A003-1), reactive oxygen species (ROS, #E004), glutathione peroxidase (GSH-Px, #A005), total antioxidant capacity (T-AOC, #A015), catalase (CAT, #A007-1) and total superoxide dismutase (T-SOD, #A001) were from (Jiancheng Bioengineering Institute, Nanjing China). Apoptosis detection kit annexin V-FITC/PI (#KGA-108) was supplied by (Jiangsu KGI, Biotech, CO., Ltd. China). A test kit for the bicinchoninic acid assay was provided by Thermo Fisher Scientific (Waltham, MA, USA). Chemiluminescence WesternBright ECL substrate kit (# ab65623) was supplied by (Abcam, Shanghai, Trading, Co., Ltd. China). The primary antibodies, heme oxygenase-1 (HO-1, #A1346), glutamate-cysteine ligase catalytic subunit (GCLC, #A1038), quinone oxidoreductase 1 (NQO1, #A1518), nuclear factor erythroid 2-related factor 2 (Nrf2, #A0674), Bcl-2-associated X protein (Bax, #A19684), caspase-3 (#A2156) and B-cell lymphoma 2 (Bcl-2, A0208) were procured from (Abclonal Tech, Woburn, MA, USA). Secondary antibodies, anti-mouse IgG (#4410), anti-rabbit IgG (#4414), and β-actin (#3700), were obtained from (Cell Signaling Technology, Boston, MA, USA).

### 2.2. Animals

Four weeks old male C57BL/6 mice were bought from Wuhan University (Wuhan, China). As an adoption period, mice were housed in separate cages and given an appropriate environment one week before the start of the experiment. Standard feed pellets and freshwater were accessible to the animal’s ad libitum. All mice were housed under laboratory conditions, light-dark period (12 h light/12 h dark), relative humidity of 45–60%, and the temperature of 22 ± 2 °C. Huazhong Agricultural University permitted the animal experiments under the Laboratory Animals Care and Ethics Committee (Permit No. HZAUMO-2018-07). Besides this, the health of experimental mice was closely monitored, and necessary measures were taken to assure the maximum welfare of the animals.

### 2.3. Experimental Design and Treatment

The 48 mice were randomly assigned into four groups as follows: (*n* = 12):

- Group 1, (CON) received physiological saline.
- Group 2, (AFB_1_), treated with 0.75 mg/kg BW aflatoxin B_1_.
- Group 3, (LUTN), treated with 50 mg/kg BW luteolin.
- Group 4, (AFB_1_ + Luteolin), treated with 0.75 mg/kg BW aflatoxin B_1_ + 50 mg/kg BW luteolin.

LUTN and AFB_1_ were dissolved in phosphate buffer saline. Following our preliminary experiment and the results of previous researchers, we chose a dose of 0.75 mg/kg BW for AFB_1_ as reported this dose could induce hepatotoxicity [28], and oral gavage of 50 mg kg BW LUTN could ameliorate liver damage in mice [29]. The experiment lasted for 15 days, and all groups received oral administration once a day at 9.00 a.m. During the whole experiment, feed intake and body gain were recorded.

### 2.4. Sample Collection

The mice were individually weighed and euthanized at the end of the experiment to collect the blood and liver samples. The serum was separated from blood samples following the centrifugation and was kept at −20 °C for serum biochemical assays. The liver tissues were removed and rinsed in ice-cold isotonic saline. Afterward, the liver samples were weighed and fixed in 4% fresh paraformaldehyde for histopathological analysis or quickly frozen in liquid nitrogen and kept at −80 °C for further assessment. The remaining liver tissues were utilized to prepare single-cell suspension for flow cytometry investigations. The following formula was used to calculate the liver coefficient:

(1) $$Liver\;coefficient\;\left(\%\right)=\frac{Liver\;weight\;\left(g\right)}{Mice\;weight\;\left(g\right)}\times 100$$

### 2.5. Determination of Serum Biochemical Indicators

An automatic biochemistry analyzer (Beckman Synchron CX4 PRO, Fullerton, CA, USA) was used to quantify the amounts of globulin and albumin, as well as GGT, ALP, AST, and ALT in serum samples following the manufacturer’s suggested protocol.

### 2.6. Hematoxylin and Eosin (H&E) Staining

The H&E staining was conducted as reported in our prior study [30]. Liver specimens were fixed for 24 h in 4% fresh paraformaldehyde solution, dried with alcohol solvent, and then embedded. The 5 µm fragments were sectioned and processed for H&E staining to assess the pathological observation in the liver of mice under a microscope (Nikon, Tokyo, Japan).

### 2.7. Determination of Oxidative Stress Indices

The 10% tissue homogenates were prepared from collected liver samples following our previously reported procedure [6]. The bicinchoninic acid determination kit was used to measure the protein concentration of tissue homogenate. ROS, MDA, T-SOD, GSH-Px, CAT and T-AOC were detected using commercially available ELISA kits. The measurements were carried out following the kit’s protocols.

### 2.8. Apoptosis Assay by Flow Cytometry

Single-cell suspensions were prepared to detect the apoptosis rate in the liver of mice following our previously described procedure [31,32]. Briefly, an annexin V-FITC/PI apoptosis detection kit was used to determine the apoptosis rate in the liver following the manufacturer’s recommended instructions. The cells were stained with annexin V-FITC (5 µL) and PI (5 µL) in the dark for 30 min at room temperature. Finally, apoptosis rates were determined using flow cytometry (Beckman-CytoFLEX Coulter, CA, USA). The data were examined by using FlowJo (BD Biosciences, NJ, USA).

### 2.9. Quantitative Real-Time PCR (qRT-PCR) and Western Blotting Analysis

The transcription levels of pertaining genes used in the present study were determined by qRT-PCR following the method previously mentioned in our study [1]. The primers tested in the current research are presented in Supplementary Table S1. Relative mRNA expression was normalized to the CON group. The 2^−ΔΔCt^ formula was used to quantify with the β-actin as a reference gene [33]. The protein expression of Nrf2 signaling and mitochondrial apoptotic pathways in mouse liver was evaluated by Western blot according to the previously described procedure [34,35]. The bicinchoninic acid assay kit was used to quantify the protein contents of samples. Chemiluminescence WesternBrightTM ECL substrate kit was used to identify the blots, and then FluroChem FC2 Imaging System was used to visualize and quantify the results.

### 2.10. Statistical Analysis

The experimental data were analyzed for significance by using SPSS (version 22., IBM Corporation, Armonk, NY, USA) software. A one-way analysis of variance (ANOVA) was used for statistical analysis, followed by the least significant difference (LSD) test. The results are presented as mean ± SD. The significance level of data was set at *p*-value < 0.05.

## 3. Results

### 3.1. Luteolin Alleviates Growth Retardation of Mice Induced by AFB_1_

**Figure 1:**
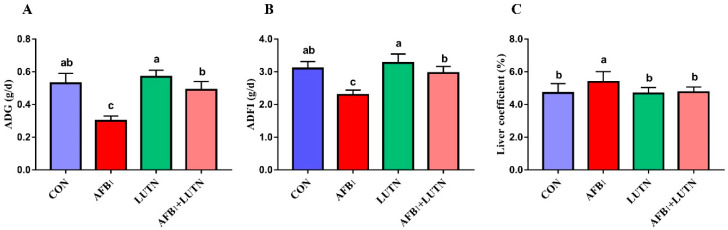
Luteolin treatment alleviates growth retardation of mice induced by aflatoxin B_1_. (**A**) Average daily gain (ADG), (**B**) average daily feed intake (ADFI) and (**C**) liver coefficients (%). The results are presented as mean ± SD *(n* = 12). ^a–c^ Columns with different lowercase letters indicated significant differences between the compared groups (*p* < 0.05).

The protective effects of luteolin (LUTN) on the growth of mice exposed to aflatoxin B_1_ (AFB_1_) are depicted in Figure 1. During the entire experimental period, the group exposed to AFB_1_ recorded the (*p* < 0.05) lowest ADG and ADFI in the comparison of CON, LUTN and AFB_1_ + LUTN groups, respectively. Contrastingly, LUTN therapy considerably improved growth performance in mice, as evidenced by increased ADG and ADFI (*p* < 0.05) compared to the AFB_1_ group. Furthermore, LUTN treatment significantly reduced liver coefficient (*p* < 0.05) increased by AFB_1_ (Figure 1C). These findings indicated that LUTN could eliminate the harmful effect of AFB_1_ on the growth of mice.

### 3.2. Luteolin Protects AFB_1_-Induced Liver Damages in Mice

**Figure 2:**
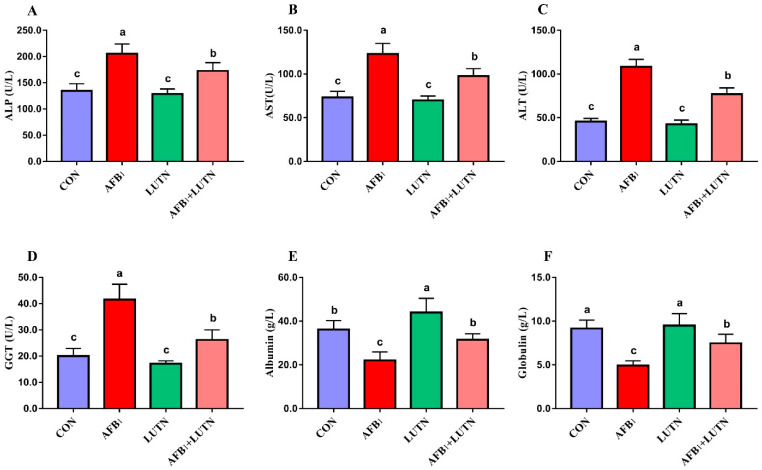
Luteolin treatment prevents AFB_1_-induced liver damages in mice. (**A**) alkaline phosphate (ALP), (**B**) aspartate aminotransferase (AST), (**C**) alanine aminotransferase (ALT) (**D**) gamma-glutamyl transferase (GGT), (**E**) albumin and (**F**) globulin. The results are presented as mean ± SD (*n* = 6). ^a–c^ Columns with different lowercase letters indicated significant differences between the compared groups (*p* < 0.05).

**Figure 3:**
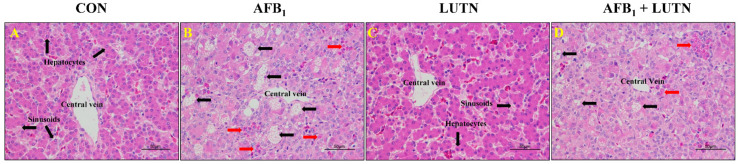
The histopathology of liver sections was stained with H&E staining. (**A**,**C**) Histological section of the liver from CON and LUTN group showed a normal histoarchitecture consisting of central vein surrounded by hepatocytes possessed sinusoids spaces. (**B**) The liver section from the AFB_1_ group showed microvesicular (black arrow) appearance of the abundant fatty droplets with small and large area of blood infiltration (red arrow) showed a hepatotoxicity. (**D**) Liver tissue from the group of mice treated with LUTN and challenged with AFB_1_ manifested recovered status of the liver from hepatotoxicity, as depicted small and rare patches of the lipids (black arrow) and the blood infiltration (red arrow) as compared to the AFB_1_ alone challenged group.

The effects of LUTN treatment on the biochemical profile of mice exposed to AFB_1_ are summarized in Figure 2A–F. Compared with the CON group, AFB_1_ exposure considerably (*p* < 0.05) increased serum liver enzymes activities such as ALP, ALT, AST, and GGT, while decreased globulin and albumin content. On the other hand, LUTN treatment considerably reversed AFB_1_-induced alterations on the biochemical profile of mice. Moreover, histological analysis revealed that AFB_1_ exposure damaged the liver structure of mice, as evident by microvesicular appearance of the lipid droplets with small and large area of blood infiltration were observed in the AFB_1_ treated group (Figure 3B). Strikingly, LUTN treatment evidently (*p* < 0.05) ameliorated and restored liver injury induced by AFB_1_, indicating that LUTN could protect the liver from AFB_1_-induced hepatotoxicity.

### 3.3. Luteolin Ameliorates Oxidative Damage in the Liver of Mice Induced by AFB_1_

**Figure 4:**
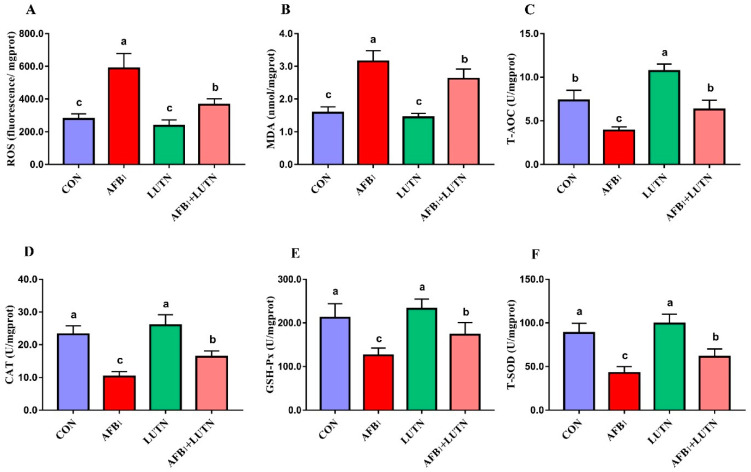
Luteolin treatment ameliorates oxidative damage in the liver of mice induced by AFB_1_. (**A**) Reactive oxygen species (ROS), (**B**) malondialdehyde (MDA) (**C**) total antioxidant capacity (T-AOC), (**D**) catalase (CAT), (**E**) glutathione peroxidase (GSH-Px) and (**F**) total superoxide dismutase (T-SOD). The results are presented as mean ± SD (*n* = 6). ^a–c^ Columns with different lowercase letters indicated significant differences between the compared groups (*p* < 0.05).

To detect the redox status in the liver of experimental mice, ROS, MDA, CAT, T-SOD, GSH-Px, and T-AOC were detected. As shown in Figure 4A,B, mice exposed to AFB_1_ revealed a significant (*p* < 0.05) increase in ROS and MDA levels. At the same time, the activities of T-AOC, CAT, GSH-Px and T-SOD were significantly reduced as compared to the CON, LUTN and AFB_1_ + LUTN groups, respectively. Contrastingly, LUTN treatment significantly (*p* < 0.05) reversed AFB_1_-induced alterations in the oxidative stress markers and antioxidant variables in the liver of mice, as evident by decreased ROS and MDA levels by 38% and 20%, respectively, while increased antioxidant enzyme activities of CAT, T-SOD, GSH-Px, and T-AOC by 36.85, 30.27, 27.26, and 40% respectively, as compared to the AFB_1_ treated group (Figure 4A–F).

### 3.4. Luteolin Treatment Prevents AFB_1_-Induced Apoptosis in Mice Hepatocytes

**Figure 5:**
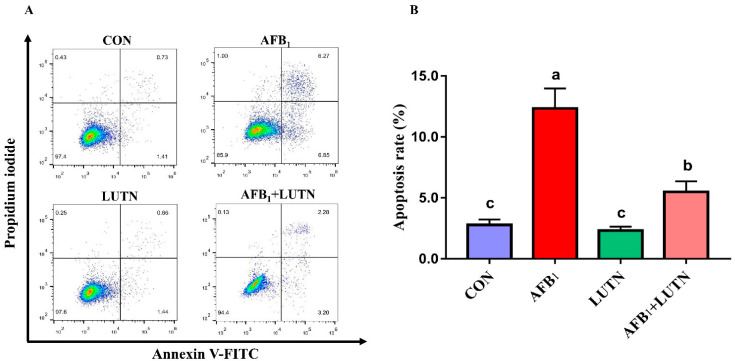
Luteolin treatment prevents AFB_1_-induced apoptosis in mice hepatocytes. (**A**) The apoptosis rates of hepatocytes were measured using flow cytometry. (**B**) Statistical analysis of apoptosis rate. The results are presented as mean ± SD (*n* = 3). ^a–c^ Columns with different lowercase letters indicated significant differences between the compared groups (*p* < 0.05).

Apoptosis rate in the hepatocytes was measured by flow cytometry (Figure 5). Apoptosis analysis revealed that AFB_1_ exposure considerably increased (*p* < 0.05) the proportion of apoptotic cells relative to the CON, LUTN, and AFB_1_ + LUTN groups, respectively. However, LUTN administration dramatically reduced (*p* < 0.05) the proportion of apoptotic cells in comparison to the AFB_1_ group.

### 3.5. Luteolin Restrains AFB_1_-Induced Mitochondrial Apoptosis Pathway

**Figure 6:**
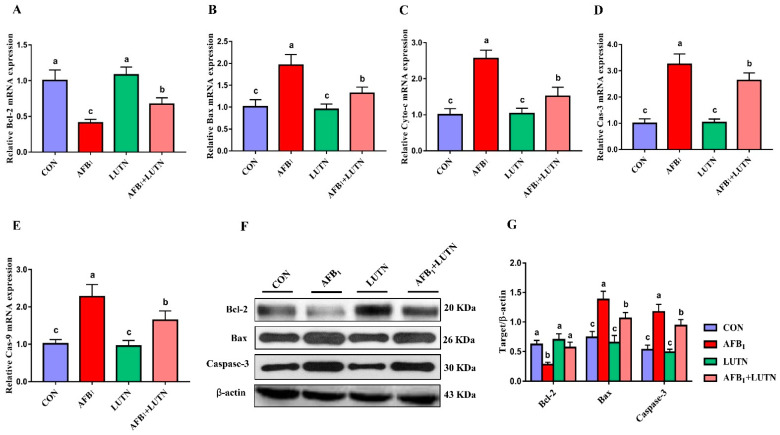
Luteolin treatment restrains AFB_1_-induced mitochondrial-mediated apoptosis pathway. The relative expression of mitochondrial-mediated apoptosis pathway transcripts was analyzed by qRT-PCR. (**A**–**E**) The relative mRNA expression of Bcl-2, Bax, Cyto-c, Cas-3 and Cas-9. The results are presented as mean ± SD (*n* = 6). Western blotting was used to detect the expression of mitochondrial apoptosis-associated proteins. (**F**) Western blotting analysis of Bcl-2, Bax and Cas-3. (**G**) Quantitative analysis of western blotting for Bcl-2, Bax and Cas-3. The results are presented as mean ± SD (*n* = 3). ^a–c^ Columns with different lowercase letters indicated significant differences between the compared groups (*p* < 0.05). B-cell lymphoma 2 (Bcl-2); Bcl-2-associated X protein (Bax); cytochrome-c (Cyt-c); caspase-3 (Cas-3); caspase-9 (Cas-9).

Mitochondrial apoptosis-associated transcription and protein expressions were detected by qRT-PCR and western blotting. As depicted in Figure 6A–E, the transcripts levels of Bax, cytochrome-c, caspase-3, and caspase-9 were significantly (*p* < 0.05) increased, while Bcl-2 was decreased under AFB_1_ exposure. Contrastingly, LUTN administration significantly reversed the transcripts levels of these genes as comparative to the AFB_1_ exposed group. Moreover, we investigated the protein expression of Bcl-2, Bax and caspase-3 in the liver tissue of mice by western blotting (Figure 6F,G). Interestingly, the results of western blotting for Bcl-2, Bax and caspase-3 were consistent with the qRT-PCR results. Similarly, in the AFB_1_ exposed group, Bcl-2 protein expression was (*p* < 0.05) down-regulated, while Bax and caspase-3 protein expression was (*p* < 0.05) up-regulated as compared to the CON group. Conversely, LUTN treatment significantly alleviated the alterations in the Bcl-2, Bax and caspase-3 protein levels induced by AFB_1_.

### 3.6. Luteolin Treatment Activates Nrf2 Signaling Pathway in the Liver of AFB_1_ Exposed Mice

**Figure 7:**
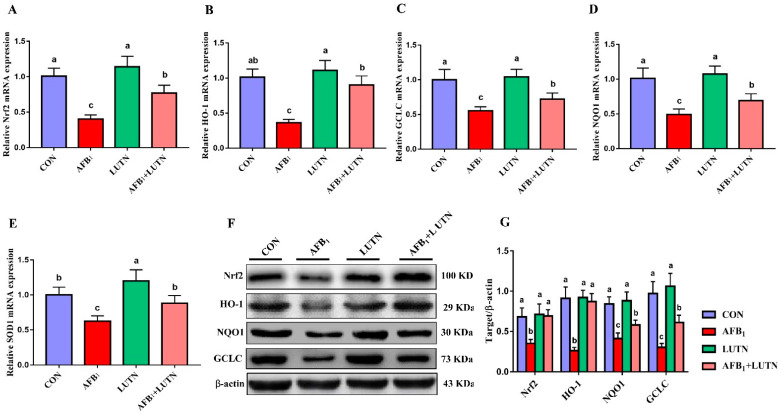
Luteolin treatment activates the Nrf2 signaling pathway in the liver of AFB_1_ exposed mice. The relative expression of Nrf2-mediated antioxidant signaling transcripts was analyzed by qRT-PCR. (**A**–**E**) The relative gene expression of Nrf2 and its downstream molecules, HO-1, GCLC, NQO1 and SOD1. The results are presented as mean ± SD (*n* = 6). The expression of Nrf2 and its associated proteins was detected by western blotting. (**F**) Western blots for Nrf2, HO-1, GCLC, and NQO1. (**G**) Quantification of western blots for Nrf2, HO-1, GCLC, and NQO1. The results are presented as mean ± SD (*n* = 3). ^a–c^ Columns with different lowercase letters indicated significant differences between the compared groups (*p* < 0.05). Nuclear erythroid-2-related factor (Nrf2); glutamate-cysteine ligase catalytic subunit (GCLC), heme oxygenase-1 (HO-1); superoxide dismutase 1 (SOD1); quinone oxidoreductase 1 (NQO1).

To confirm our hypothesis that LUTN promotes the antioxidant capacity and alleviates hepatotoxicity in mice induced by AFB_1_ is associated with Nrf2 signaling pathway activation, the transcript levels and protein expression of Nrf2 and downstream targets were detected. As depicted in Figure 7A–E, AFB_1_ exposed group showed a significant (*p* < 0.05) decrease in the gene expression of Nrf2, HO-1, GCLC, NQO1 and SOD1 compared to the CON and other experimental groups. In contrast, LUTN treatment considerably improved the transcript expressions of Nrf2, HO-1, GCLC, NQO1 and SOD1 altered by AFB_1_ (*p* < 0.05). Further, the protein expression of Nrf2 and its target genes were detected by western blotting and revealed the same tendency (Figure 7F,G). Similarly, protein expression of Nrf2 and its downstream targets, including HO-1, GCLC, and NQO1, were (*p* < 0.05) down-regulated in the AFB_1_ challenged group. However, LUTN treatment considerably up-regulated the Nrf2, NQO1, HO-1 and GCLC protein expressions as down-regulated by AFB_1_ (*p* < 0.05).

## 4. Discussion

Aflatoxins, threatening mycotoxins, are commonly found in cereals and animal forages and pose major health and economic risks to humans and animals [36]. Growth retardation is one of the most important symptoms of aflatoxins poisoning. In the present study, AFB_1_ exposure decreased ADG and ADFI in mice. The observed growth retardation may have resulted from anorexia, suppression of lipogenesis, and protein synthesis induced by AFB_1_ [37]. The liver is regarded as the main target organ of AFB_1_ poisoning. We found that AFB_1_ generated clinical and pathological symptoms of liver injury in mice, as evident from the increased haptic enzymes AST, ALT, ALP, GGT, and decreased globulin and albumin content as well as fatty droplets and hepatocytes infiltration with macro vesicles in the liver of mice. These findings imply that AFB_1_ can cause hepatotoxicity, consistent with previous findings [28,38,39]. Interestingly, luteolin (LUTN) treatment attenuated growth retardation and alleviated toxic effects on serum biochemical profile and pathological changes in the liver of mice induced by AFB_1_. The current results align with a previous study, which has reported that LUTN supplementation prevented acetaminophen-induced hepatic injury in rats [16]. The present findings strongly suggested that LUTN treatment can mitigate AFB_1_-induced hepatic damage in mice. However, special clinical studies are warranted to know the hepatoprotective effects of LUTN on ongoing or established AFB_1_ toxicity in humans or animals.

Oxidative stress is considered to be critical molecular process underlying cell damage [40]. Oxidative stress is associated with significant increase in the generation of reactive oxygen species (ROS) while decreased antioxidant capacity in the body [41]. Previous studies reported that excessive ROS production is a significant cause of AFB_1_-induced hepatotoxicity and apoptosis in mice [5,30,42]. Similarly, in the present study, AFB_1_ exposure generated ROS and MDA accumulation and inhibited antioxidant enzyme activities such as T-SOD, CAT, GAH-Px and T-AOC, and induced apoptosis in the liver of mice. LUTN is considered a potent ROS scavenger, protecting cells from ROS accumulation and apoptosis induced by oxidative stress [43]. We found that LUTN treatment substantially suppressed oxidative stress induced by AFB_1_ as evidenced by decreased ROS and MDA accumulation, strengthened antioxidant defense system (T-SOD, CAT, GAH-Px and T-AOC), and reduced apoptosis rate in the liver of mice.

Mitochondria have been identified as the primary targets for AFB_1_ harmful effects in causing apoptosis [44]. The mitochondrial apoptosis pathway is regulated by anti-apoptotic and pro-apoptotic members of the Bcl-2 family [45]. Bcl-2 is an anti-apoptotic protein that impedes the release of apoptogenic molecules (Cyt-c), while Bax is a pro-apoptotic protein that promotes the release of Cyt-c into the cytoplasm by competing with Bcl-2, thereby causing cell death [46,47]. The release of Cyt-c into the cytoplasm, resulted in caspase-3 and caspase-9 activation and the induction of apoptosis [48,49]. Previously, studies reported that AFB_1_ exposure could cause apoptosis in various tissues and cells and the mechanism was linked with the mitochondrial apoptosis pathway [5,50,51,52]. Our results showed that expressions of Bax, Cyt-c caspase-3 and caspase-9 were considerably up-regulated, while Bcl-2 was down-regulated by AFB_1_. These findings demonstrated that AFB_1_-induced excessive apoptosis in the liver of mice is linked with the mitochondrial apoptosis pathway. Notably, LUTN treatment increased the Bcl-2 expression, inhibited mitochondrial Cyt-c release and reduced the activated caspase-3 and caspase-9 expression in the liver of mice under AFB_1_ exposure. Furthermore, mitochondria are the primary target of ROS attacks, and superfluous ROS production can lead to oxidative stress and mitochondrial malfunctioning [31]. We observed that LUTN suppressed ROS generation induced by AFB_1_. As a result, we hypothesized that LUTN reduces AFB_1_-induced excessive apoptosis in mouse liver either directly or through the suppression of oxidative stress.

Nrf2 is a transcription factor that plays a significant role in the process of AFB_1_-induced cytotoxicity [53,54]. Under normal conditions, Nrf2 stays in the cytosol by its specific antagonist, Keap1, while under stimulation, it dissociates from Keap1 and translocates to the nucleus, where it binds to ARE and regulates the transcripts of downstream antioxidant genes (HO-1, NQO1, SOD, GCLC) [55]. Nrf2 and its targeted antioxidative genes, HO-1, NQO1, SOD, and GCLC, are critical components to maintain the redox system and have been shown to exhibit cytoprotective resistance against oxidative stress [56]. The present study revealed that AFB_1_ exposure inhibited the expression of Nrf2 and its associated-target genes such as HO-1, NQO1, SOD1, and GCLC in the liver of mice. However, LUTN dramatically rescued these effects induced by AFB_1_. Previously, LUTN prevents the progression of liver fibrosis induced by carbon tetrachloride-(CCL4) through targeting AKT/mTOR/p70S6K and TGFβ/Smad signaling pathways [26]. The current results agree with previous investigations that AFB_1_ exposure suppressed Nrf2 nuclear translocation [28,54], and LUTN could rescue cells from oxidative damage by activating Nrf2 and up-regulating the cellular antioxidant genes expression [57,58,59]. However, further research is needed to address the preventive effects of LUTN against ongoing and/or established AFB_1_-induced toxicity in human and/or animal.

## 5. Conclusions

**Figure 8:**
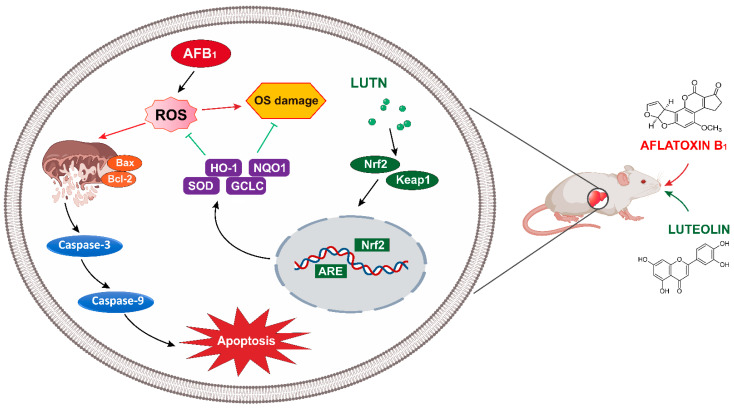
Schematic diagram representing hepatoprotective mechanism of luteolin against AFB_1_-induced apoptosis and oxidative stress via the activation of Nrf2 signaling pathway in the liver of mice.

The current study provides significant evidence on the potential protective effects of luteolin (LUTN) against AFB_1_-induced hepatotoxicity in mice. LUTN effectively rescued liver injury, as evident by the amelioration of toxic effects on serum biochemical profile and pathological alterations induced by AFB_1_. LUTN attenuates AFB_1_-induced excessive apoptosis by inhibiting the mitochondria-dependent apoptosis pathway. Additionally, LUTN suppressed AFB_1_-induced oxidative stress by scavenging ROS accumulation and enhancing antioxidant enzymes capacity via regulation of Nrf2 signaling. The current study suggested that the key mechanisms underlying the LUTN hepatoprotective effects were associated with the activation of the Nrf2 signaling pathway (Figure 8). The present study suggested that LUTN may serve as a potential mitigator against AFB_1_-induced liver injury and could be helpful for the development of novel treatment to combat liver diseases in humans and/or animals.

## Data Availability

Data is contained within the article and supplementary materials.

## Supplementary Materials

The following are available online at https://www.mdpi.com/article/10.3390/antiox10081268/s1, Table S1: List of primers used in the present study for qRT-PCR analysis.
