# Supplementary material for: Luteolin Alleviates AflatoxinB1-Induced Apoptosis and Oxidative Stress in the Liver of Mice through Activation of Nrf2 Signaling Pathway
Source: Antioxidants (Basel). 2021 Aug 9;10(8):1268. doi: 10.3390/antiox10081268 (PMC8389199; doi:10.3390/antiox10081268)
Supplement: Supplementary file 1 [file antioxidants-10-01268-s001.zip › antioxidants-1311556-supplementary.pdf]

**Table S1.** Sequence of the primers used for quantitative real-time PCR assay.

| Target gene | Primer | Primer sequence (5′→3′) | Accession No. |
|---|---|---|---|
| Bax | Forward<br>Reverse | GAGCTGCAGAGGATGATTGCT<br>TGATCAGCTCGGGCACTTTA | NM_007527.3 |
| Bcl-2 | Forward<br>Reverse | CTCGTCGCTACCGTCGTGACTTCG<br>CAGATGCCGGTTCAGGTACTCAGTC | NM_009741.3 |
| Caspase-3 | Forward<br>Reverse | GCTTGGAACGGTACGCTAAGA<br>CCCAGAGTCCACTGACTTGC | NM_010295.2 |
| Caspase-9 | Forward<br>Reverse | CTGCCAAGAAAATGGTCACGG<br>ACACGGAGCATCCATCTGTC | NM_001277932.1 |
| Cyto-C | Forward<br>Reverse | AAGGGAAGACGATGACGG<br>TGAGAAACCGCTCCACATA | NM_010849 |
| Nrf2 | Forward<br>Reverse | ATGACCATGAGTCGCTTGCC<br>AATCAGTCATGGCTGCCTCC | NM_010902.4 |
| HO-1 | Forward<br>Reverse | TTGCCAGTGCCACCAAGTTC<br>TCAGCAGCTCCTGCAACTCC | NM_001014912.1 |
| NQO1 | Forward<br>Reverse | GGTGAGCTGAAGGACTCGAA<br>ACCACTGCAATGGGAACTGAA | NM_008706.5 |
| GCLC | Forward<br>Reverse | AAGATGCGGAGGCATCAAA<br>TCAAAGCCATAACAATTGGGCAG | NM_010295.2 |
| SOD1 | Forward<br>Reverse | GGAACCATCCACTTCGAGCA<br>CTGCACTGGTACAGCCTTGT | NM_011434.1 |
| β-actin | Forward<br>Reverse | GTTGGAGCAAACATCCCCCA<br>ACGCGACCATCCTCCTCTTA | NM_007397.5 |
